# A free energy perturbation-assisted machine learning strategy for mimotope screening in neoantigen-based vaccine design

**DOI:** 10.1093/bib/bbaf254

**Published:** 2025-07-10

**Authors:** Qinglu Zhong, Kevin C Chan, Lei Fu, Ruhong Zhou

**Affiliations:** College of Life Sciences, College of Physics, Institute of Quantitative Biology, Zhejiang University, 866 Yuhangtang Road, Hangzhou 310058, China; Shanghai Institute for Advanced Study, Zhejiang University, 799 Dangui Road, Shanghai 201203, China; College of Life Sciences, College of Physics, Institute of Quantitative Biology, Zhejiang University, 866 Yuhangtang Road, Hangzhou 310058, China; Shanghai Institute for Advanced Study, Zhejiang University, 799 Dangui Road, Shanghai 201203, China; College of Life Sciences, College of Physics, Institute of Quantitative Biology, Zhejiang University, 866 Yuhangtang Road, Hangzhou 310058, China; Shanghai Institute for Advanced Study, Zhejiang University, 799 Dangui Road, Shanghai 201203, China; College of Life Sciences, College of Physics, Institute of Quantitative Biology, Zhejiang University, 866 Yuhangtang Road, Hangzhou 310058, China; Shanghai Institute for Advanced Study, Zhejiang University, 799 Dangui Road, Shanghai 201203, China; Zhejiang Key Laboratory of Cell and Molecular Intelligent Design and Development, Zhejiang University, 866 Yuhangtang Road, Hangzhou 310058, China; Department of Chemistry, Columbia University, New York, NY 10027, United States

**Keywords:** free-energy perturbation, machine learning, neoantigen-based vaccine, mimotope, Bayesian optimization

## Abstract

Neoantigen-based immunotherapy has emerged as a promising approach for cancer treatment. One key strategy in neoantigen-based vaccine design is to alter known neoantigens into enhanced mimotopes that elicit more robust immune responses. However, screening mimotopes presents challenges in both diversity and precision. While machine learning (ML) models facilitate high-throughput screening of immunogenic candidates, they struggle to distinguish mimotopes from original neoantigens (i.e. identify mimotopes with higher binding affinities, rather than solely distinguish between binding and nonbinding peptides). In contrast, alchemical methods such as free energy perturbation (FEP) provide quantitative binding free-energy differences between mimotopes and neoantigens but are computationally intensive. To leverage the strengths of both approaches, we propose an FEP-assisted ML (FEPaML) strategy that employs Bayesian optimization to iteratively refine knowledge-based predictions with physics-based evaluations, thereby progressively achieving locally optimized, precise, and robust outcomes. Our FEPaML strategy is then applied to screen mimotopes for several representative neoantigens. It has demonstrated excellent predictive precisions (exceeding 0.9) with a relatively small number of FEP samplings, significantly outperforming existing ML models.

## Introduction

According to the World Health Organization, cancer is the second leading cause of death globally [1]. Cancer immunotherapy, as the fourth leading treatment of cancer after surgery, chemotherapy and radiotherapy, has gained growing attention in recent years. Neoantigens, the core of cancer immunotherapy, are immunogenic peptides derived from aberrant proteins exclusively expressed by tumor cells, accompanied by tumor-specific mutations [2]. These neoantigens can be presented by human leukocyte antigen (HLA) proteins on the surface of tumor cells, where they are recognized as foreign (nonself) components by T-cell receptors (TCR), thereby triggering adaptive immune responses to eliminate tumor cells [3]. Consequently, neoantigens become promising targets [4] for cancer immunotherapy on account of their inherent tumor-specificity. Neoantigen-based vaccines, specifically peptide vaccines in this study, have shown potential to elicit and amplify robust anti-tumor immune responses as well as to provide long-term control of cancers [5]. Several neoantigen-based vaccines have been evaluated in clinical trials [4, 6] for diverse tumor types, such as melanoma [7, 8] and glioblastoma [9, 10]. These vaccines offer advantages in terms of high specificity, low toxicity, cost-effectiveness, and ease of production [4]. However, natural neoantigens are often relatively weak ligands that are insufficient to trigger a robust immune response [11].

An effective scheme for enhancing neoantigen-based vaccine efficacy is to alter known neoantigens into enhanced mimotopes [12, 13]. These mimotopes are designed by substituting or mutating residues to improve binding affinity towards HLA or TCR, thereby acting as effective mimics to trigger similar immune responses [14–17]. Although several enhanced mimotopes have been successfully translated into clinical trials for cancer vaccines [18–22], the diversity and precision required for mimotope screening pose significant challenges.

The diversity challenge is reflected in the individual-specific responses to immunotherapy [23]. Each individual’s unique genetic makeup leads to the expression of different immune proteins, resulting in thousands of variations in HLA diversity worldwide [24] and millions of variations in TCR diversity for each individual [25]. Stochastic genetic mutations further contribute to the diversity of neoantigens and their mimotope candidates among individuals. Neither immunological experiments nor computational simulations can feasibly address such extraordinary diversity and identify universally effective neoantigens. Fortunately, machine learning (ML) excels at handling such big data. Numerous ML platforms [26], such as NetMHCpan [27], MHCflurry [28], TransPHLA [29], can predict immunogenicity for millions of peptides within a limited time while maintaining an adequate level of accuracy. Consequently, ML has become an indispensable tool for preliminary identification or screening of neoantigens [30].

On the other hand, the precision challenge arises because ML platforms often struggle to distinguish mimotopes from original neoantigens due to their closely resembling ML features. However, immunological experiments and computational simulations have the ability to precisely distinguish between such resembling entities. Molecular dynamics (MD) simulation has been widely used to simulate and compute thermodynamic properties of complex biomolecular systems [31], enabling various free energy calculation methods. Among these methods, a class of rigorous physics-based methods known as free energy perturbation (FEP) specialize in quantifying binding free energy difference between a ligand and its analog [32]. This is achieved by focusing the “perturbation” on the minor change from a ligand to its analog. FEP methods have been demonstrated to be the most consistent with experimental results [32], and have been successfully applied to protein-ligand studies in drug discovery [33] and vaccine design [34], despite their computational intensity.

To address the challenges of both diversity and precision in mimotope screening, we propose an FEP-assisted ML (FEPaML) strategy that integrates the efficiency of ML with the precision of FEP methods. In this strategy, ML predictions will be iteratively refined by FEP samplings through Bayesian optimization [35, 36] to progressively achieve locally optimized and precise predictions of the research interest. In the proposed ML model, we designed a dual-loss mechanism to balance between a large amount of knowledge-based rough data sourced from public database and a limited quantity of physics-based precise data obtained through FEP samplings. Our strategy demonstrated its feasibility and effectiveness by significantly improving the predictive precision of screening mimotopes for the hot-spot neoantigen p53^R175H^, surpassing the performance of prevalent ML platforms. This strategy offers a promising approach to the design of neoantigen-based vaccines for further clinical trials.

## Methods

### Overview of workflows

**Figure 1:**
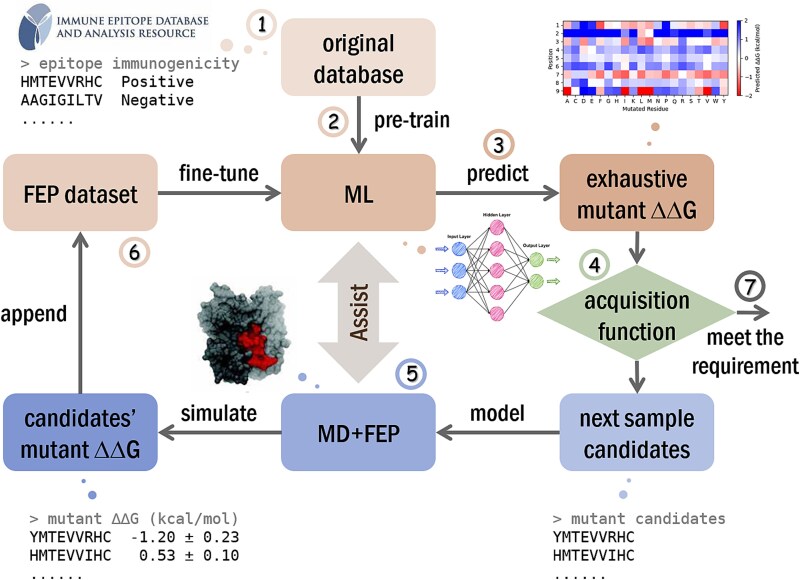
Workflows of FEPaML strategy.

FEPaML is a strategy for efficient and precise screening of enhanced mimotopes (mutants) with higher binding affinity towards HLA than the natural neoantigen (original). This strategy is illustrated in Fig. 1, which includes seven steps.

Step 1: **Data selection**. Retrieve peptides (epitopes) of desired length and specific HLA from Immune Epitope Database (IEDB). The selected dataset typically contains thousands of positive or negative immunogenicity records, indicating whether the epitopes bind to the specific HLA or not.

**Figure 2:**
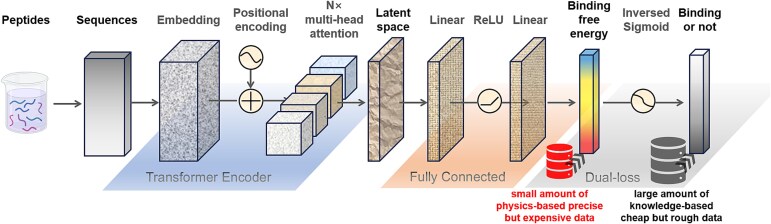
Framework of the ML model. The length, width, and height of each cuboid denote sequence length, dimension of features, and batch size, respectively. The black text in the top row denotes entities and the gray text denotes functions.

Step 2: **Pretraining**. Use the above dataset for preliminary training of a ML model. Epitope sequences serve as the inputs, and immunogenicity labels serve as the outputs. A Transformer Encoder [37] is used as the base, followed by a fully connected layer appended to project the latent space to binding free energies. Finally, an inversed Sigmoid layer is appended to transform these binding free energies to binary outputs (Fig. 2).

Step 3: **ML prediction**. After preliminary training, the ML model predicts the binding free energy ∆*G* for any peptide to the specific HLA, then compute the difference between the mutant and the original to obtain the mutant relative binding free energy, ∆∆*G* = ∆*G_mutant_*-∆*G_original_*.

Step 4: **Candidates election**. Define an acquisition function [36] based on a desired objective (e.g. 100-fold increase in binding affinity), to evaluate the gap and direction between the current prediction and the desired objective. Elect candidates from mutant samples that align with the optimization direction for further precise sampling.

Step 5: **FEP calculation**. Calculate the relative binding free energy ∆∆*G* of the above mutant candidates using MD+FEP and append the results to the FEP dataset. MD+FEP calculates the free energy change ∆*G* from the original to the mutant, considering both unbound and HLA-bound states. This mutant relative binding free energy ∆∆*G* = ∆*G_bound_*-∆*G_unbound_* should be equivalent to the predicted ∆∆*G* in Step 3 in theory, as free energy is a state function independent of the calculation path.

Step 6: **Fine-tuning**. Use the updated FEP dataset to fine-tune the pretrained ML model. The fine-tuning architecture is identical to that in the pre-training process, except with a dual-loss mechanism to balance the small amount of precise FEP data and the large amount of IEDB binary data.

Step 7: **Iterative optimization**. Iterate steps 3–6 until the desired objective is met, following the Bayesian optimization algorithm (described below) [35, 36].

### Bayesian optimization

In Bayes Theorem [38], $P\left(A|B\right)=P(A)\cdot P\left(B|A\right)/P(B)$, where *P* denotes the probability of an event, event *A* or event *B*. *P*(*A*|*B*) means posterior probability, which is updated probability after the evidence (event *B*) is considered. *P*(*A*) means prior probability, which is the probability before the evidence is considered. *P*(*B*|*A*) means likelihood, which is the probability of the evidence given the belief (event *A*) is true. *P*(*B*) means marginal probability, which is the probability of the evidence under any circumstances. The Bayesian view provides a way to revise existing predictions given additional evidence, in other words, updating the prior distribution using posterior information. For the proposed FEPaML strategy, the ML predictions in Step 3 can be regarded as the prior *P*(*A*), and the updated ML predictions given additional FEP data in Step 6 can be regarded as the posterior *P*(*A*|*B*). The belief *A* can be replaced by ML, and the evidence *B* can be replaced by FEP. The Bayes Theorem becomes $P\left( ML| FEP\right)=P(ML)\cdot P\left( FEP| ML\right)/P(FEP)$. After *n* round iterations, the updated posterior *P*(*ML_n_*|*FEP_n_*), which also serves as the next round’s prior *P*(*ML_n+1_*), can be calculated as $P\left(M{L}_{n+1}\right)=P\left(M{L}_n\right)\cdot P\left( FE{P}_n|M{L}_n\right)/P\left( FE{P}_n\right)$. This indicates that the next ML predictions (*ML_n+1_*) are only dependent on the previous ML predictions (*ML_n_*) and the previous FEP data (*FEP_n_*). FEP data is necessary and sufficient for ML predictions to become more accurate through Bayesian optimization iteration. Assuming that FEP is more precise than ML, the ML predictions must eventually converge to the ground truth independent of FEP data, $P\left(M{L}_{n+1}\right)=P\left(M{L}_n| FE{P}_n\right)\approx P\left(M{L}_n\right)$. The convergence destination and convergence efficiency are determined by the user-defined acquisition function, which acts as the decision-maker of Bayesian optimization. Two primary opposing strategies, exploitation (Depth-First-Search, DFS) and exploration (Breadth-First-Search, BFS), will be discussed in Results. Different acquisition strategies [36] will result in different convergence destinations with different efficiency.

### Machine learning algorithm

The ML framework is illustrated in Fig. 2. The framework refers to the BERT model [39] solving natural language processing problems. Here, epitope sequences are considered as natural language, where individual residues are treated as input tokens. In addition, a [CLS] token [39] is added at the beginning of each sequence to represent the overall feature of the epitope. The vocabulary of the embedding dictionary becomes 21, consisting of 20 types of natural amino acids and a [CLS] token. After embedding layer, a standard positional encoding [37] layer is added to inject information about the relative or absolute position of the tokens in the sequence. *N*× standard multihead self-attention layers are appended to learn the latent space of the sequence. These elements constitute a standard Transformer Encoder. The latent space of [CLS] is propagated into a fully connected layer to project onto the binding free energy corresponding to the epitope. According to the Boltzmann distribution, the binding free energy is proportional to the negative logarithm of the ratio of bound to unbound probability, $\varDelta G\propto -{k}_{\mathrm{B}}T\cdot \log \frac{P_{bound}}{P_{unbound}}$. For IEDB binary labels, strong negative binding free energies correspond to positive labels, while positive or less strong negative binding free energies correspond to negative labels (there may be a constant difference related to the binding threshold, but it does not affect the subsequent content here in ML). The binding free energy regression therefore can be translated into binary classification by an inversed Sigmoid function.

For pretraining procedure, the model input with IEDB binary dataset is trained through five-fold cross-validation. The optimal parameters are as follows: a dimension of 32, four multihead, one attention layer, a batch size of 1024, and a learning rate of 1e-4. The loss function here is the normal binary cross-entropy loss. The pretraining procedure is continued until the Area Under the Curve (AUC) of the validation set reaches convergence.

For fine-tuning procedure, both IEDB binary dataset and FEP dataset are input to the model. Two different types of datasets/labels require two different types of loss functions. The loss function for IEDB binary dataset remains as the binary cross-entropy loss, while that for FEP dataset adopts the mean-square-error loss. The total loss is defined as a linear combination of these two losses, $Los s= Los{s}_{\mathrm{BCE}}\left(\mathrm{IEDB}\right)+k\cdot Los{s}_{\mathrm{MSE}}\left(\mathrm{FEP}\right)$. The parameter *k* has no explicit significance since these two types of losses are not comparable. Moreover, the two losses are imbalanced in value that *Loss*_BCE_ has been minimized during pretraining while *Loss*_MSE_ has not. In order to balance the two types of losses and steadily optimize the total loss, we propose a self-adaptive dual-loss mechanism. For each epoch, *k* will be updated to the present value of *Loss*_BCE_ / *Loss*_MSE_, if greater than the present *k*. As a result, *k* will steadily increase within a defined range from 0.0001 to 1 during the fine-tuning procedure. The learning rate decreases layer-by-layer backwards from the last layer in the fine-tuning procedure. The other parameters remain the same as those in the pretraining procedure.

### Molecular dynamics simulations

The crystal structure of the neoantigen-HLA binding complex under investigation was obtained from the Protein Data Bank (PDB). The missing residues or missing atoms of PDB structures were completed using MODELLER [40]. The complex was positioned into a 10 nm cubic box and dissolved with TIP3P [41] water molecules. Then, 150 mM NaCl (sodium and chloride ions) were added to mimic the physiological condition and neutralize the net charge of the system. MD simulations were performed using GROMACS [42] 2020.6 with CHARMM36 force field [43]. The system was subjected to energy minimization using the steepest descent algorithm, then gradually heated to 310 K and equilibrated under constant pressure. The step size of leapfrog Verlet integrator was set to 2 fs. Particle-Mesh Ewald method [44] was utilized to treat the long-range electrostatic interactions, while a smooth cutoff (with a cutoff distance of 1.2 nm) was chosen for treating the short-range van der Waals interactions. Linear Constraint Solver (LINCS) algorithm [45] was used to constrain covalent bonds involving hydrogen atoms. The Nose–Hoover Thermostat [46, 47] was applied to couple temperature at 310 K in the system every 2 ps. The Parrinello–Rahman Barostat [48] was used to control pressure in the system at 1 bar. The production run was performed for 100 ns to ensure the system reached equilibrium.

### Free-energy perturbation

FEP methods have been widely used in mutagenesis studies to calculate relative binding free energies of protein-ligand complexes based on MD simulations [34, 49, 50]. The binding free energy of a ligand to a protein is $\varDelta G={G}_{complex}-{G}_{ligand}-{G}_{protein}$. The binding free energy difference between ligand A and ligand B to the same protein is $\varDelta \varDelta G=\varDelta{G}_B-\varDelta{G}_A=\left({G}_{complex B}-{G}_{complex A}\right)-\left({G}_{ligand B}-{G}_{ligand A}\right)=\varDelta{G}_{complex}-\varDelta{G}_{ligand}$. Both Δ*G_complex_* (bound state) and Δ*G_ligand_* (unbound state) can be calculated by perturbing from ligand A to ligand B through an alchemical pathway [51], wherein a parameter *λ* is employed to describe the gradual transformation from ligand A to ligand B, ranging from 0 to 1. The free energy difference can be integrated using Zwanzig relationship [52] $\varDelta G={\sum}_{\lambda}\varDelta{G}_{\lambda }={\sum}_{\lambda }-{\beta}^{-1}\ln{\left\langle \exp \left[-\beta \left({U}_{\lambda +\varDelta \lambda}-{U}_{\lambda}\right)\right]\right\rangle}_{\lambda }$, where *U* denotes the potential energy of the system and $U_{\lambda}=(1-\lambda)U_{A}+\lambda U_{B} $. Furthermore, considering that the free energy integrated through the forward and the backward pathway should be equivalent, the multistate Bennett acceptance ratio estimator (MBAR) [53] is employed as a more robust algorithm for FEP calculations.

In this study, the hybrid structures and topologies of the natural neoantigen and its mimotopes were generated using the pmx [54] tool. Soft-core potentials [55] were used for nonbonded interactions. The other options were the same as those mentioned in the previous section. Each FEP calculation was assigned to 16 *λ* windows, then performed for 3 ns per *λ* window using GROMACS. The relative binding free energy calculation for each mimotope was performed for a total of 288 ns (3 ns × 16 *λ* windows × 3 replicas × bound & unbound state), then analyzed by MBAR algorithm using the Alchemical Analysis [56] tool.

### p53^R175H^ neoantigen

In this study, we first selected p53^R175H^ neoantigen as a representative model system (more examples below) to demonstrate the proposed strategy for mimotope screening. TP53, which encodes the tumor suppressor protein p53 [57], is the most frequently mutated driver gene across all cancer types [58]. TP53 mutations are common across different cancer patients [59], therefore some immunogenic p53 mutants have been identified as shared neoantigens, which might be promising targets for personalized vaccine design that would be shared among a broader range of patients [60]. The Catalog of Somatic Mutations in Cancer database (https://cancer. sanger.ac.uk) shows that arginine-to-histidine at position 175 (R175H) is the most frequent mutation in p53. The neoantigen was identified as residues 168–176 of p53^R175H^ (HMTEVVRHC) [61, 62], which was presented by the specific HLA allele HLA-A*02:01.

### Datasets

We collected a dataset of 57 340 9-mer peptides with binary labels indicating whether they can be presented by HLA-A*02:01 from IEDB, after removing duplicate and inconsistent data. This dataset is denoted as IEDB binary dataset in the context. Among which, we further extracted a subset of 5843 9-mer peptides with experimentally determined binding affinity to HLA-A*02:01. This subset is denoted as IEDB binding affinity dataset in the context. The aforementioned datasets were collected before 31 December 2021 and mainly served as training sets. We used two recently published datasets as test sets, which were obtained from the ongoing peptide-HLA binding prediction contest [63] organized by IEDB (http://tools.iedb.org/auto_bench/mhci/weekly). Contest 0803 dataset (*n* = 196) refers to the dataset published at 3 August 2023 with IEDB reference 1 042 685. Contest 0810 dataset (*n* = 107) refers to the dataset published at 10 August 2023 with IEDB reference 1 042 751. We exhaustively calculated the relative binding free energy using MD+FEP for all possible single-site mutations (*n* = 171) of the neoantigen p53^R175H^, and collected them as a test dataset, denoted as p53-mut FEP dataset in the context.

### Prediction models

Our proposed FEPaML model has several versions to be discussed in the context. FEPaML_PT refers to the model pretrained on the IEDB binary dataset. FEPaML_BA refers to the model fine-tuned with the IEDB binding affinity dataset. FEPaML_ALA refers to the model fine-tuned with 9 p53-mut FEP data sampled by alanine scanning. FEPaML_DFS.25 refers to the model fine-tuned with 25 p53-mut FEP data sampled by DFS (more below). FEPaML_DFS+B4.32 refers to the model fine-tuned with 32 p53-mut FEP data sampled by DFS+B4 (more below). For comparison, we referred to three well-established peptide-HLA binding prediction models: NetMHCpan [27], MHCflurry [28], TransPHLA [29]. NetMHCpan_EL and NetMHCpan_BA refer to eluted ligand score and binding affinity score in NetMHCpan-4.1 [27]. MHCflurry_PS and MHCflurry_BA refer to presentation score and binding affinity score in MHCflurry-2.0 [28]. TransPHLA refers to binding probability in TransPHLA [29]. NetMHCpan_EL, MHCflurry_PS, TransPHLA, and FEPaML_PT are mainly applicable for predicting binding or nonbinding since they are trained only on binary data. The remaining models are applicable for predicting binding affinity.

### Metrics

The performance of ML predictions in this study was evaluated using four metrics, AUC, SRCC, Precision, and Recall. AUC and SRCC are the official metrics for IEDB contests. AUC, which stands for area under the receiver operating characteristic curve, is primarily used for evaluating the performance of binary classification. SRCC, which stands for Spearman’s rank correlation coefficient, is primarily used for evaluating the performance of regression. AUC and SRCC evaluate the performance of all samples, whereas Precision and Recall focus on the performance of positive samples. Precision measures the ratio of true positives to all predicted positives, while Recall measures the ratio of true positives to all actual positives. Precision and Recall, originally used for binary classification, can be extended to regression analysis [64].

The focus of this study is to identify mimotopes with higher binding affinity (i.e. lower binding free energy) than the natural neoantigen. This implies us to classify samples with ΔΔ*G* < 0 as positives and the others as negatives. A Scale Function *φ* is defined to map ΔΔ*G* onto the [0,1] scale similar to binary classification,


$$ \varphi (y)=\frac{1}{1+{\exp}^{s\left(g-c\right)}} $$

where *c* and *s* describe the center and the shape of the function. A Hit Function *α* is defined to assess the accuracy of the predicted ΔΔ*G*,


$$ \alpha \left(\hat{y},y\right)=\mathcal{I}\left(\left|\hat{y}-y\right|\leqslant \varepsilon \right)\cdot \left(1-\exp \left[-k\frac{{\left(\left|\hat{y}-y\right|-\varepsilon \right)}^2}{\varepsilon^2}\right]\right) $$

where *k* determines the shape of the function, *ε* is the threshold of the deviation between the predicted label $\hat{y}$ and the actual label *y*, *I* is the indicator function given 1 if its argument is true and 0 otherwise. We defined *ε* = 1 kcal/mol, *c* = 0 kcal/mol, *s* = 5, *k* = 10 in this study.


$$ Precision=\frac{\sum_i\alpha \left({\hat{y}}_i,{y}_i\right)\cdot \varphi \left({\hat{y}}_i\right)}{\sum_i\varphi \left({\hat{y}}_i\right)} $$

$$ Recall=\frac{\sum_i\alpha \left({\hat{y}}_i,{y}_i\right)\cdot \varphi \left({y}_i\right)}{\sum_i\varphi \left({y}_i\right)} $$

## Results

### Machine learning model pretrained with IEDB binary dataset

**Figure 3:**
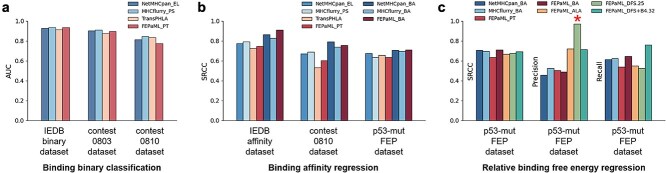
Performance of FEPaML model compared with other well-established models. (a) AUC measures the performance of binding binary classification. Detailed ROC curves in Fig. S1. (b) SRCC measures the performance of binding affinity regression. Detailed datapoints in Fig. S2. (c) SRCC, precision and recall measure the performance of relative binding free energy regression. Datasets and prediction models are described in Methods.

Our proposed ML model is an allele-specific peptide-HLA (pHLA) binding predictor, which offers several advantages over pan-specific [27–29] models. Allele-specific models require less training data, as the data are specific to a particular HLA allele. They also eliminate the need for allele information in the input, thereby reducing the model size and computational requirements. Additionally, our ML model can be trained from scratch, allowing for better focus on the task-related training dataset. The ML model was pre-trained on our selected IEDB binary dataset, following the procedures outlined in Methods. We evaluated the performance of the model on three datasets, including this IEDB binary dataset and two test datasets from pHLA binding prediction contest [63]. Our pre-trained model exhibited comparable performance to well-established pHLA binding prediction programs, such as NetMHCpan [27], MHCflurry [28], TransPHLA [29] (Figs 3a and S1). Overall, the AUC of binary classification reached approximately 0.9, which is a generally acknowledged performance among prevalent programs. These results demonstrate the feasibility of our task-related pre-training approach. However, it is important to note that mimotope screening requires the ability to distinguish pHLA binding with higher affinities, rather than solely distinguish between binding and non-binding peptides. This limitation motivates the need for further refinement of our ML model using more specialized data on binding affinity.

### ML model fine-tuned with Immune Epitope Database binding affinity dataset

In order to improve the ML model for predicting pHLA binding affinities, our pretrained model was then fine-tuned on our selected IEDB affinity dataset, following the procedures outlined in Methods. We evaluated the performance of this fine-tuned model on three datasets, including this IEDB affinity dataset, a contest dataset, and our p53-mut FEP dataset. The SRCC of our binding affinity predictions were 0.90, 0.76, and 0.71 for these datasets, respectively (Figs 3b and S2). Our fine-tuned model exhibited comparable performance to NetMHCpan and MHCflurry, both of which had been trained using binding affinity data. In contrast, the preceding four models tested in binary classification exhibited a relatively lower level of performance, since they had never learned from binding affinity data. These results suggest that models trained on more “specialized” data have the capability to differentiate more “specialized” events, thereby improving predictive performance within the “specialized” domain. However, it is important to recognize that these general binding affinity data may still lack the necessary specialization for mimotope screening, which requires precise discrimination of mimotopes with higher binding affinity from the natural neoantigen. This limitation suggests the need for further refinement of the ML model using more specialized data relevant to the particular neoantigen.

### Precision and recall are more suitable metrics for mimotope screening

**Figure 4:**
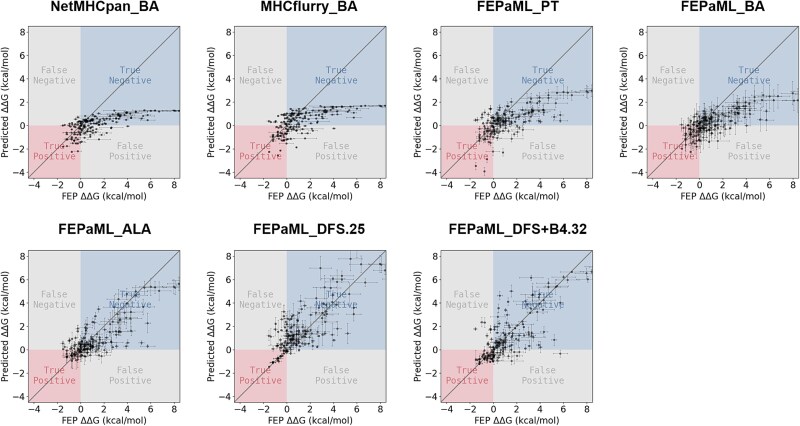
ML prediction versus FEP calculation of relative binding free energy of p53^R175H^ mimotopes. These results correspond to the performance in Fig. 3c.

The specialized data for mimotope screening is supposed to include data on analogs of the natural neoantigen, such as single-site mutagenesis data. We attempted to fine-tune our ML model using a simple single-site mutagenesis dataset, containing relative binding free energies of alanine-scanning p53^R175H^ mutants calculated by FEP. However, the SRCC for the p53-mut FEP dataset did not show improvement compared to those generalized models (Fig. 3c). This observation suggests that SRCC may not be a suitable metric for evaluating the models in the context of mimotope screening. SRCC is a global metric that assesses the overall correlation between predicted and actual values, but mimotope screening primarily focuses on identifying mutants with higher binding affinities. The focus lies in accurately identifying true positive samples, without necessarily considering the performance on true negative samples (Fig. 4).

To address this issue, we propose using precision and recall as more suitable metrics for mimotope screening. Precision measures the proportion of true positives among all predicted positives, reflecting the quality of the designed vaccines. Recall, on the other hand, measures the proportion of true positives among all actual positives, indicating the quantity of identified vaccine candidates. Our ML model, fine-tuned with alanine-scanning data, demonstrated a significant improvement in precision compared to those models trained on general binding affinity data (Fig. 3c). These results suggest that incorporating even more “specialized” mutagenesis data can further improve the predictive performance of ML model for the “specialized” task. Although incorporating alanine-scanning mutagenesis dataset has demonstrated efficacy, we remain uncertain about the comparative effectiveness of other alternative mutagenesis strategies. Considering the resource-intensive nature of constructing a comprehensive specialized mutagenesis dataset from scratch, developing an efficient strategy for data sampling becomes a critical challenge to be addressed.

### More efficient sampling strategy during Bayesian optimization

In order to efficiently search for valid data samples, we incorporated Bayesian optimization algorithm into our FEPaML strategy. Mathematically, the optimization goal is to find minima *x* of the *Model*(*x*) within a given local space. For mimotope screening, our goal becomes to identify mutant samples with maximum binding affinity, i.e. most negative ΔΔ*G*. Bayesian optimization poses this as a problem in sequential decision theory, where a decision-maker iteratively makes a decision based on the feedback from the previous decision to optimize the model as quickly as possible.

Bayesian optimization has two primary opposing strategies: exploitation (DFS) and exploration (BFS), which are governed by an acquisition function serving as the decision-maker. The DFS strategy focuses on electing the nonrepetitive mutant with the minimal binding free energy from the previous ML prediction for the next FEP sampling, similar to the Greedy Algorithm. In contrast, the BFS strategy emphasizes exploring mutants by replacing the residue at each position with the recommended residue from the previous ML prediction for the next FEP sampling. Alanine scanning is a typical example of BFS. To optimize the high costs associated with direct BFS, we combined DFS with a variable Breadth *n* (DFS+B*n*): candidates for next FEP sampling will include the DFS candidate and its *X*-scanning within the range of *n* neighboring positions, where *X* is the mutated residue of the DFS candidate.

**Figure 5:**
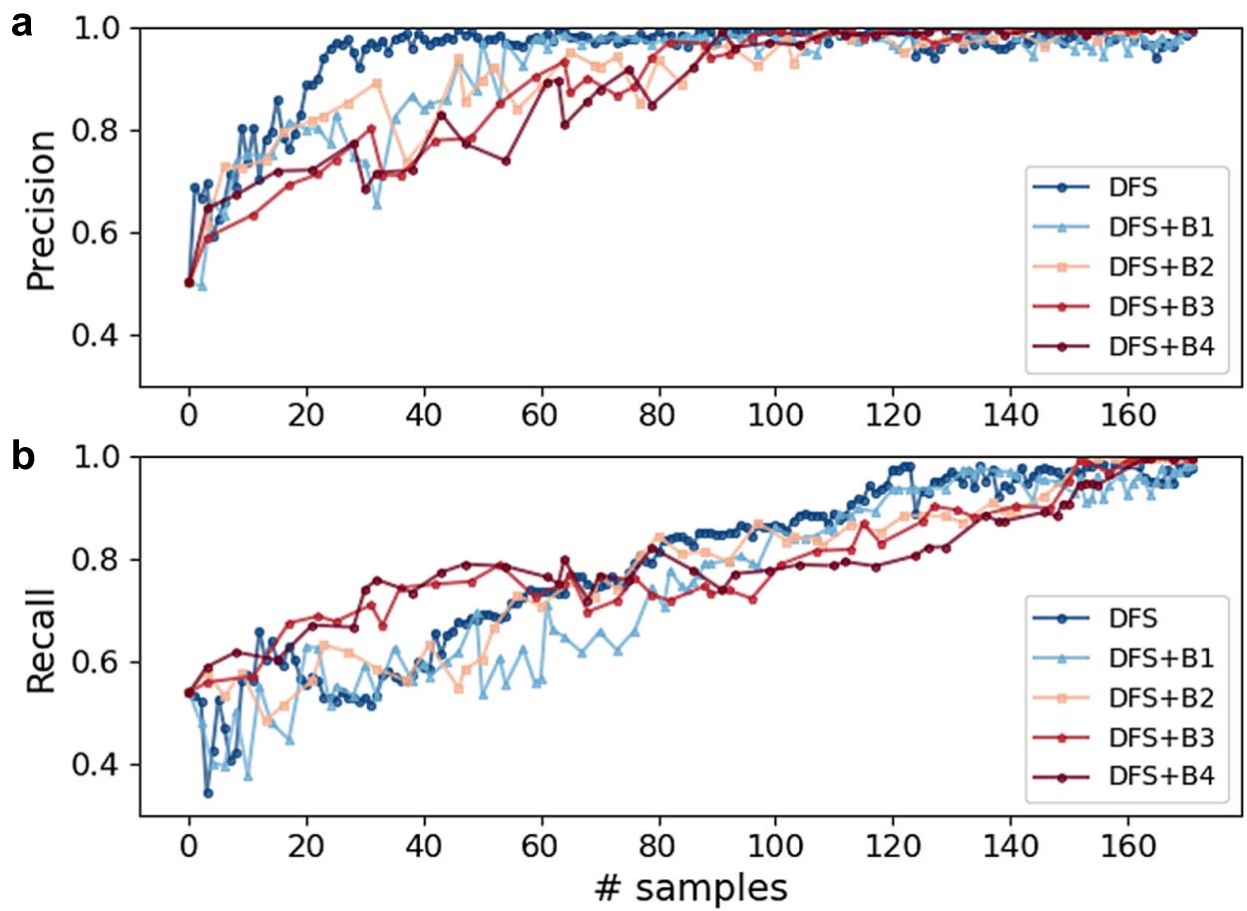
(a) Precision and (b) recall on p53-mut FEP dataset. X-axis is the number of FEP samples used for fine-tuning the ML model along with Bayesian optimization iteration. DFS+B*n* refers to Depth-First-Search combined with search breadth of *n*.

**Figure 6:**
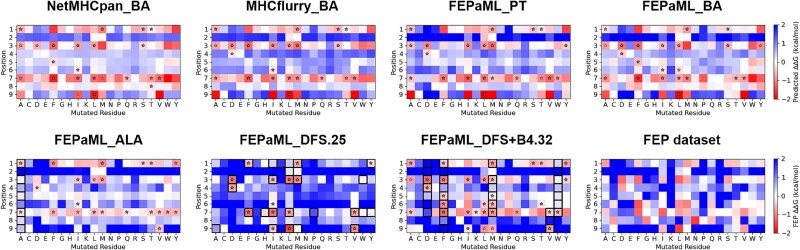
The relative binding free energy of exhaustive single-site mutants based on the p53^R175H^ neoantigen predicted by ML models (the first seven) and calculated by FEP (the last one). The star markers refer to the true positive samples. The pixel-level black boxes refer to the FEP samples for fine-tuning. These results correspond to the performance in Figs 3c and 4.

The optimization efficiency was evaluated using DFS+B*n* strategies, with *n* ranging from 0 to 4. The results showed that a narrower sampling strategy (e.g. DFS) leads to accelerated enhancement of predictive precision (Fig. 5a), while a broader sampling strategy (e.g. DFS+B4) results in faster improvement in predictive recall, but with slower convergence (Fig. 5b). The implementation of the DFS strategy significantly enhanced predictive precision (Fig. 5a), achieving a precision of 0.9 after fine-tuning the ML model with just 25 FEP samples (FEPaML_DFS.25 in Figs 3c and 4). FEPaML_DFS.25 significantly reduced the occurrence of false positives compared to FEPaML_PT, while maintaining a similar number of true positives, thereby enhancing the precision (Figs 4 and 6). On the other hand, the implementation of the DFS+B4 strategy quickly improved predictive recall (Fig. 5b), achieving a recall of 0.76 after fine-tuning the ML model with 32 FEP samples (FEPaML_DFS+B4.32 in Figs 3c and 4). FEPaML_DFS+B4.32 significantly increased the number of true positives compared to FEPaML_PT, but with an accompanying increase in false positives (Figs 4 and 6). Considering that different strategies lead to distinct optimization directions, the choice of sampling strategy should be guided by the desired trade-off between precision and recall, tailored to the specific design objectives. We further conducted an ablation analysis to assess the contribution of the generalized IEDB dataset and the specialized FEP dataset to this optimal performance. The results demonstrated that both datasets are indispensable (Fig. S3). Due to the limited availability of specialized FEP data, ablating generalized IEDB data could easily lead to overfitting. While emphasizing the importance of specialized data, we also recognize that a robust model necessitates generalized data to build a solid foundation.

### The general applicability of the FEP-assisted ML strategy

We employed two additional examples to evaluate the generalization performance of the FEPaML strategy. The first example is the immunogenic peptide SLLMWITQC derived from cancer-testis antigen NY-ESO-1 [65, 66], and the second one is the immunogenic peptide RMFPNAPYL derived from Wilms Tumor transcription factor WT1 [67, 68]. We used the same procedures as those applied for p53^R175H^ to screen mimotopes for these two peptides, arriving at similar conclusions. Initially, the predictive precision of a generalized ML model was below 0.6. However, after fine-tuning the model with about 25 FEP samplings acquired through DFS, the predictive precision significantly improved to over 0.9 (Figs S4 and S5). These results demonstrate the superiority and general applicability of the FEPaML strategy in enhancing the precision of mimotope screening for potential vaccine candidates. The requirement for a relatively small number of FEP samplings (around 25) highlights the efficiency of the proposed strategy, saving computational resources with assistance from specialized data acquisition.

## Discussions

The importance of specialized high-quality data in ML-based molecular design strategies cannot be overstated. Our ML model fuses a tiny set of “specialized” and precise binding free energy data into a large amount of “generalized” and rough binary immunogenicity data using a dual-loss mechanism, which adaptively balances these multimodal data. Our results demonstrated that fine-tuning ML models with a set of specialized mutagenesis data could significantly enhance predictive precision, effectively addressing the limitations of generalized models in evaluating mimotopes (Fig. 3c). In contrast, various generalized models trained on homologous data yielded comparable performance (Fig. 3a and b). This finding aligns well with the current trend in ML development, which emphasizes a prioritized importance [69] of specialized high-quality data over sophisticated algorithms, as reflected in the shift from “Attention is all you need” [37] to “Textbooks are all you need” [70].

We employ Bayesian optimization to acquire the specialized high-quality data from scratch. Bayesian optimization is intrinsically a mathematical optimization algorithm used to efficiently search for optimal enhanced candidates within an extensive pool of possible mimotopes. A self-defined acquisition function is used to determine the optimization directions towards enhancing binding affinity, serving as the decision-maker for diverse sampling strategies. Our results also highlight the “contrast” between the DFS and BFS strategies. DFS focuses on intensively searching around the current optimal candidate, leading to faster but potentially premature convergence to local optima, while neglecting other potential candidates. On the other hand, BFS emphasizes exploring a broader range of candidates, resulting in a slower convergence and a higher resource consumption, but with the capability to discover rare but better (global optima) candidates. Considering that vaccine design is supposed to prioritize quality and screening efficiency rather than quantity, DFS can be regarded as a superior sampling strategy for screening vaccine candidates. However, for other extended applications, the data sampling strategy needs to be determined based on their specific research objectives.

We discussed mostly on single-mutant mimotope screening in this work. For multi-mutant mimotopes, the optimization space will expand exponentially. We propose treating multi-site mutations as multiple single-site mutations, altered one by one along Bayesian optimization iterations with either DFS or BFS. The synergy among multi-site mutations is expected to be inferred through the ML model, independent of the mutation order.

The FEPaML strategy for mimotope screening still has some limitations that need to be addressed in future studies. In this work, we focused only on mimotope-HLA binding affinity, which is a prerequisite for immunogenicity but still requires specific TCR recognition and subsequent immune processes. However, the performance of current generalized HLA-epitope-TCR binding prediction models [71–74] remains far below that of generalized HLA-epitope binding prediction models shown in Fig. 3a. This indicates that existing generalized HLA-epitope-TCR data are insufficient to capture the complexity of TCR recognition. Some studies further suggest that the affinity between TCR and HLA-epitope should not be excessively high [75, 76]. Current ML models still struggle to address this challenge associated with TCR recognition or even cross-reactivity. Our strategy highlights the importance of specialized high-quality data, and have the potential to enhance local prediction precision of mimotope screening based on specific HLA-epitope-TCR cases in future studies.

Although our strategy demonstrates high performance in precision and efficiency, its transferability across diverse epitopes remains limited. This is because our strategy primarily aims to optimize the ML model towards local precision on demand, rather than enhancing cross-epitope transferability. Achieving both high precision and broad transferability would require more diverse specialized datasets in future studies.

FEP results always contain inherent errors, but these errors have not been effectively utilized in our strategy yet. Uncertainty quantification becomes crucial for improving ML model reliability [77]. Since FEP errors represent physical uncertainty, they are valuable to be incorporated into our ML model (e.g. through Evidential [78] or Bayesian [35] neural networks) to better quantify prediction uncertainty in future studies. This would enable more informed decision-making under ambiguity and enhance trustworthiness in mimotope screening.

In this work, we propose an FEPaML strategy that integrates the advantages of highly efficient ML and precise MD+FEP methods. ML, as a data-driven or knowledge-based approach, excels when abundant knowledge is available but struggles when data are limited. On the other hand, MD+FEP, as a physics-based approach, simulates systems under the laws of physics regardless of existing knowledge. In the context of neoantigen-based vaccine design, the available data often seem insufficient due to the vast diversity of immunogenic molecules. By integrating both knowledge-based and physics-based approaches through Bayesian optimization, our strategy updates the knowledge-based prior distribution using physics-based posterior information. The application of this strategy for screening vaccine candidates based on several representative neoantigens resulted in a significant improvement in predictive precision with notable efficiency. This idea holds potential for further applications to generalized peptide design [79] or even other biotechnological fields, where data diversity and precision play critical roles. The successful integration of multisource data in this strategy offers a promising framework for tackling similar challenges encountered in imbalanced multimodal data fusion [80, 81]. This work builds a bridge between the imperfect The Fourth Paradigm [82] and the previous conventional paradigms for scientific explorations.

**Key Points**

- This work highlights the critical role of high-quality task-specific data in improving the performance of ML models for neoantigen-based vaccine design.
- Our proposed FEPaML strategy effectively integrates the efficiency of ML and the precision of FEP, addressing the challenges of both diversity and precision in mimotope screening.
- By incorporating a small amount of customized high-quality FEP data into a large amount of generic data from IEDB, our strategy significantly enhances predictive precision with notable efficiency in mimotope screening.

## Supplementary Material

SuppData_bbaf254

## Code availability

Source code is available at https://github.com/zhongqinglu/FEPaML

## References

[1] Sung H, Ferlay J, Siegel RL. et al. Global cancer statistics 2020: GLOBOCAN estimates of incidence and mortality worldwide for 36 cancers in 185 countries. *CA Cancer J Clin* 2021;71:209–49. 10.3322/caac.21660 33538338

[2] Jiang T, Shi T, Zhang H. et al. Tumor neoantigens: from basic research to clinical applications. *J Hematol Oncol J Hematol Oncol* 2019;12:93. 10.1186/s13045-019-0787-5 31492199 PMC6731555

[3] Sim MJW, Sun PD. T cell recognition of tumor Neoantigens and insights into T cell immunotherapy. *Front Immunol* 2022;13:833017. 10.3389/fimmu.2022.833017 35222422 PMC8867076

[4] Xie N, Shen G, Gao W. et al. Neoantigens: promising targets for cancer therapy. *Signal Transduct Target Ther* 2023;8:9. 10.1038/s41392-022-01270-x 36604431 PMC9816309

[5] Shetty K, Ott PA. Personal Neoantigen vaccines for the treatment of cancer. *Annu Rev Cancer Biol* 2021;5:259–76. 10.1146/annurev-cancerbio-060820-111701

[6] Biswas N, Chakrabarti S, Padul V. et al. Designing neoantigen cancer vaccines, trials, and outcomes. *Front Immunol* 2023;14:1105420. 10.3389/fimmu.2023.1105420 36845151 PMC9947792

[7] Ott PA, Hu Z, Keskin DB. et al. An immunogenic personal neoantigen vaccine for patients with melanoma. *Nature* 2017;547:217–21. 10.1038/nature22991 28678778 PMC5577644

[8] Hu Z, Leet DE, Allesøe RL. et al. Personal neoantigen vaccines induce persistent memory T cell responses and epitope spreading in patients with melanoma. *Nat Med* 2021;27:515–25. 10.1038/s41591-020-01206-4 33479501 PMC8273876

[9] Keskin DB, Anandappa AJ, Sun J. et al. Neoantigen vaccine generates intratumoral T cell responses in phase Ib glioblastoma trial. *Nature* 2019;565:234–9. 10.1038/s41586-018-0792-9 30568305 PMC6546179

[10] Hilf N, Kuttruff-Coqui S, Frenzel K. et al. Actively personalized vaccination trial for newly diagnosed glioblastoma. *Nature* 2019;565:240–5. 10.1038/s41586-018-0810-y 30568303

[11] Katsikis PD, Ishii KJ, Schliehe C. Challenges in developing personalized neoantigen cancer vaccines. *Nat Rev Immunol* 2023;24:213–27. 10.1038/s41577-023-00937-y 37783860 PMC12001822

[12] Candia M, Kratzer B, Pickl WF. On peptides and altered peptide ligands: from origin, mode of action and design to clinical application (immunotherapy). *Int Arch Allergy Immunol* 2016;170:211–33. 10.1159/000448756 27642756 PMC7058415

[13] Slansky JE, Nakayama M. Peptide mimotopes alter T cell function in cancer and autoimmunity. *Semin Immunol* 2020;47:101395. 10.1016/j.smim.2020.101395 32205022 PMC7160047

[14] Macdonald WA, Chen Z, Gras S. et al. T cell Allorecognition via molecular mimicry. *Immunity* 2009;31:897–908. 10.1016/j.immuni.2009.09.025 20064448

[15] He X, Zhou S, Quinn B. et al. Position-scanning peptide libraries as particle Immunogens for improving CD8 ^+^ T-cell responses. *Adv Sci* 2021;8:2103023. 10.1002/advs.202103023 PMC8693074 34716694

[16] Alonso JA, Smith AR, Baker BM. Tumor rejection properties of gp100209-specific T cells correlate with T cell receptor binding affinity towards the wild type rather than anchor-modified antigen. *Mol Immunol* 2021;135:365–72. 10.1016/j.molimm.2021.05.001 33990005 PMC8184619

[17] Smith AR, Alonso JA, Ayres CM. et al. Structurally silent peptide anchor modifications allosterically modulate T cell recognition in a receptor-dependent manner. *Proc Natl Acad Sci* 2021;118:e2018125118. 10.1073/pnas.2018125118 33468649 PMC7848747

[18] Ribas A, Weber JS, Chmielowski B. et al. Intra–lymph node prime-boost vaccination against Melan a and Tyrosinase for the treatment of metastatic melanoma: results of a phase 1 clinical trial. *Clin Cancer Res* 2011;17:2987–96. 10.1158/1078-0432.CCR-10-3272 21385924

[19] Romano E, Michielin O, Voelter V. et al. MART-1 peptide vaccination plus IMP321 (LAG-3Ig fusion protein) in patients receiving autologous PBMCs after lymphodepletion: results of a phase I trial. *J Transl Med* 2014;12:97. 10.1186/1479-5876-12-97 24726012 PMC4021605

[20] Filipazzi P, Pilla L, Mariani L. et al. Limited induction of tumor cross-reactive T cells without a measurable clinical benefit in early melanoma patients vaccinated with human leukocyte antigen class I–modified peptides. *Clin Cancer Res* 2012;18:6485–96. 10.1158/1078-0432.CCR-12-1516 23032742

[21] Schwartzentruber DJ, Lawson DH, Richards JM. et al. gp100 peptide vaccine and Interleukin-2 in patients with advanced melanoma. *N Engl J Med* 2011;364:2119–27. 10.1056/NEJMoa1012863 21631324 PMC3517182

[22] Chen S, Li Y, Depontieu FR. et al. Structure-based Design of Altered MHC class II–restricted peptide ligands with heterogeneous immunogenicity. *J Immunol* 2013;191:5097–106. 10.4049/jimmunol.1300467 24108701 PMC3888030

[23] Chowell D, Morris LGT, Grigg CM. et al. Patient HLA class I genotype influences cancer response to checkpoint blockade immunotherapy. *Science* 2018;359:582–7. 10.1126/science.aao4572 29217585 PMC6057471

[24] Robinson J, Waller MJ, Parham P. et al. IMGT/HLA database—a sequence database for the human major histocompatibility complex. *Nucleic Acids Res* 2001;29:210–3. 10.1093/nar/29.1.210 11125094 PMC29780

[25] Soto C, Bombardi RG, Kozhevnikov M. et al. High frequency of shared Clonotypes in human T cell receptor repertoires. *Cell Rep* 2020;32:107882. 10.1016/j.celrep.2020.107882 32668251 PMC7433715

[26] Addala V, Newell F, Pearson JV. et al. Computational immunogenomic approaches to predict response to cancer immunotherapies. *Nat Rev Clin Oncol* 2024;21:28–46. 10.1038/s41571-023-00830-6 37907723

[27] Reynisson B, Alvarez B, Paul S. et al. NetMHCpan-4.1 and NetMHCIIpan-4.0: improved predictions of MHC antigen presentation by concurrent motif deconvolution and integration of MS MHC eluted ligand data. *Nucleic Acids Res* 2020;48:W449–54. 10.1093/nar/gkaa379 32406916 PMC7319546

[28] O’Donnell TJ, Rubinsteyn A, Laserson U. MHCflurry 2.0: improved pan-allele prediction of MHC class I-presented peptides by incorporating antigen processing. *Cell Syst* 2020;11:42–48.e7. 10.1016/j.cels.2020.06.010 32711842

[29] Chu Y, Zhang Y, Wang Q. et al. A transformer-based model to predict peptide–HLA class I binding and optimize mutated peptides for vaccine design. *Nat Mach Intell* 2022;4:300–11. 10.1038/s42256-022-00459-7

[30] Lang F, Schrörs B, Löwer M. et al. Identification of neoantigens for individualized therapeutic cancer vaccines. *Nat Rev Drug Discov* 2022;21:261–82. 10.1038/s41573-021-00387-y 35105974 PMC7612664

[31] Hansson T, Oostenbrink C, Van Gunsteren W. Molecular dynamics simulations. *Curr Opin Struct Biol* 2002;12:190–6. 10.1016/S0959-440X(02)00308-1 11959496

[32] Ross GA, Lu C, Scarabelli G. et al. The maximal and current accuracy of rigorous protein-ligand binding free energy calculations. *Commun Chem* 2023;6:222. 10.1038/s42004-023-01019-9 37838760 PMC10576784

[33] Muegge I, Hu Y. Recent advances in alchemical binding free energy calculations for drug discovery. *ACS Med Chem Lett* 2023;14:244–50. 10.1021/acsmedchemlett.2c00541 36923913 PMC10009785

[34] Song Y, Bell DR, Ahmed R. et al. A mutagenesis study of autoantigen optimization for potential T1D vaccine design. *Proc Natl Acad Sci* 2023;120:e2214430120. 10.1073/pnas.2214430120 37040399 PMC10120010

[35] Ghahramani Z . Probabilistic machine learning and artificial intelligence. *Nature* 2015;521:452–9. 10.1038/nature14541 26017444

[36] Grosnit A, Cowen-Rivers AI, Tutunov R. et al. Are we forgetting about compositional optimisers in Bayesian optimisation? Journal of Machine Learning Research 2021;22:1–78.

[37] Vaswani A, Shazeer N, Parmar N. et al. Attention Is All You Need 2023.

[38] Malakoff D . A brief guide to Bayes theorem. *Science* 1999;286:1461–1. 10.1126/science.286.5444.1461

[39] Devlin J, Chang M-W, Lee K. et al. BERT: pre-training of deep bidirectional transformers for language understanding. 2019.

[40] Webb B, Sali A. Comparative protein structure Modeling using MODELLER. *Curr Protoc Bioinformatics* 2016;54:5.6.1-5.6.37. 10.1002/cpbi.3 PMC5031415 27322406

[41] Jorgensen WL, Chandrasekhar J, Madura JD. et al. Comparison of simple potential functions for simulating liquid water. *J Chem Phys* 1983;79:926–35. 10.1063/1.445869

[42] Van Der Spoel D, Lindahl E, Hess B. et al. GROMACS: fast, flexible, and free. *J Comput Chem* 2005;26:1701–18. 10.1002/jcc.20291 16211538

[43] Best RB, Zhu X, Shim J. et al. Optimization of the additive CHARMM all-atom protein force field targeting improved sampling of the backbone ϕ, ψ and side-chain χ _1_ and χ _2_ dihedral angles. *J Chem Theory Comput* 2012;8:3257–73. 10.1021/ct300400x 23341755 PMC3549273

[44] Essmann U, Perera L, Berkowitz ML. et al. A smooth particle mesh Ewald method. *J Chem Phys* 1995;103:8577–93. 10.1063/1.470117

[45] Hess B, Bekker H, Berendsen HJC. et al. LINCS: a linear constraint solver for molecular simulations. *J Comput Chem* 1997;18:1463–72. 10.1002/(SICI)1096-987X(199709)18:12<1463::AID-JCC4>3.0.CO;2-H

[46] Nosé S . A molecular dynamics method for simulations in the canonical ensemble. *Mol Phys* 1984;52:255–68. 10.1080/00268978400101201

[47] Hoover WG . Canonical dynamics: equilibrium phase-space distributions. *Phys Rev A* 1985;31:1695–7. 10.1103/PhysRevA.31.1695 9895674

[48] Parrinello M, Rahman A. Polymorphic transitions in single crystals: a new molecular dynamics method. *J Appl Phys* 1981;52:7182–90. 10.1063/1.328693

[49] Song Y, Lee S, Bell D. et al. Binding affinity calculations of gluten peptides to HLA risk modifiers: DQ2.5 versus DQ7.5. *J Phys Chem B* 2022;126:5151–60. 10.1021/acs.jpcb.2c00962 35796490

[50] Zhou H, Chan KC, Buratto D. et al. The rigidity of a structural bridge on HLA-I binding groove explains its differential outcome in cancer immune response. *Int J Biol Macromol* 2023;253:127199. 10.1016/j.ijbiomac.2023.127199 37793526

[51] Fu H, Chipot C, Shao X. et al. Standard binding free-energy calculations: how far are we from automation? *J Phys Chem B* 2023;127:10459–68. 10.1021/acs.jpcb.3c04370 37824848

[52] Zwanzig RW . High-temperature equation of state by a perturbation method. *I Nonpolar Gases J Chem Phys* 1954;22:1420–6. 10.1063/1.1740409

[53] Shirts MR, Chodera JD. Statistically optimal analysis of samples from multiple equilibrium states. *J Chem Phys* 2008;129:124105. 10.1063/1.2978177 19045004 PMC2671659

[54] Gapsys V, Michielssens S, Seeliger D. et al. Pmx: automated protein structure and topology generation for alchemical perturbations. *J Comput Chem* 2015;36:348–54. 10.1002/jcc.23804 25487359 PMC4365728

[55] Beutler TC, Mark AE, Van Schaik RC. et al. Avoiding singularities and numerical instabilities in free energy calculations based on molecular simulations. *Chem Phys Lett* 1994;222:529–39. 10.1016/0009-2614(94)00397-1

[56] Klimovich PV, Shirts MR, Mobley DL. Guidelines for the analysis of free energy calculations. *J Comput Aided Mol Des* 2015;29:397–411. 10.1007/s10822-015-9840-9 25808134 PMC4420631

[57] Liu Y, Su Z, Tavana O. et al. Understanding the complexity of p53 in a new era of tumor suppression. *Cancer Cell* 2024 S1535610824001338;42:946–67. 10.1016/j.ccell.2024.04.009 38729160 PMC11190820

[58] Sabapathy K, Lane DP. Therapeutic targeting of p53: all mutants are equal, but some mutants are more equal than others. *Nat Rev Clin Oncol* 2018;15:13–30. 10.1038/nrclinonc.2017.151 28948977

[59] Zehir A, Benayed R, Shah RH. et al. Mutational landscape of metastatic cancer revealed from prospective clinical sequencing of 10,000 patients. *Nat Med* 2017;23:703–13. 10.1038/nm.4333 28481359 PMC5461196

[60] Zhao W, Wu J, Chen S. et al. Shared neoantigens: ideal targets for off-the-shelf cancer immunotherapy. *Pharmacogenomics* 2020;21:637–45. 10.2217/pgs-2019-0184 32423288

[61] Lo W, Parkhurst M, Robbins PF. et al. Immunologic recognition of a shared p53 mutated Neoantigen in a patient with metastatic colorectal cancer. *Cancer Immunol Res* 2019;7:534–43. 10.1158/2326-6066.CIR-18-0686 30709841 PMC6685528

[62] Malekzadeh P, Pasetto A, Robbins PF. et al. Neoantigen screening identifies broad TP53 mutant immunogenicity in patients with epithelial cancers. *J Clin Invest* 2019;129:1109–14. 10.1172/JCI123791 30714987 PMC6391139

[63] Trolle T, Metushi IG, Greenbaum JA. et al. Automated benchmarking of peptide-MHC class I binding predictions. *Bioinformatics* 2015;31:2174–81. 10.1093/bioinformatics/btv123 25717196 PMC4481849

[64] Torgo L, Ribeiro R. Precision and recall for regression. *Discov Sci* 2009;5808:332–46. 10.1007/978-3-642-04747-3_26

[65] Ishihara M, Nishida Y, Kitano S. et al. A phase 1 trial of NY-ESO -1-specific TCR -engineered T-cell therapy combined with a lymph node-targeting nanoparticulate peptide vaccine for the treatment of advanced soft tissue sarcoma. *Int J Cancer* 2023;152:2554–66. 10.1002/ijc.34453 36727538

[66] Webb AI, Dunstone MA, Chen W. et al. Functional and structural characteristics of NY-ESO-1-related HLA A2-restricted epitopes and the Design of a Novel Immunogenic Analogue. *J Biol Chem* 2004;279:23438–46. 10.1074/jbc.M314066200 15004033

[67] Oji Y, Kagawa N, Arita H. et al. WT1 trio peptide-based cancer vaccine for rare cancers expressing shared target WT1. *Cancers* 2023;15:393. 10.3390/cancers15020393 36672344 PMC9857088

[68] Borbulevych OY, Do P, Baker BM. Structures of native and affinity-enhanced WT1 epitopes bound to HLA-A0201: implications for WT1-based cancer therapeutics. *Mol Immunol* 2010;47:2519–24. 10.1016/j.molimm.2010.06.005 20619457 PMC2930271

[69] Mock M, Edavettal S, Langmead C. et al. AI can help to speed up drug discovery — but only if we give it the right data. *Nature* 2023;621:467–70. 10.1038/d41586-023-02896-9 37726439

[70] Gunasekar S, Zhang Y, Aneja J. et al. Textbooks Are All You Need 2023.

[71] Chen J, Zhao B, Lin S. et al. TEPCAM : Prediction of T -cell receptor–epitope binding specificity via interpretable deep learning. *Protein Sci* 2024;33:e4841. 10.1002/pro.4841 37983648 PMC10731497

[72] Zhou Z, Chen J, Lin S. et al. GRATCR: epitope-specific T cell receptor sequence generation with data-efficient pre-trained models. *IEEE J Biomed Health Inform* 2025;29:2271–83. 10.1109/JBHI.2024.3514089 40031605

[73] Feng Z, Chen J, Hai Y. et al. Sliding-attention transformer neural architecture for predicting T cell receptor–antigen–human leucocyte antigen binding. *Nat Mach Intell* 2024;6:1216–30. 10.1038/s42256-024-00901-y

[74] Zhang Y, Wang Z, Jiang Y. et al. Epitope-anchored contrastive transfer learning for paired CD8+ T cell receptor–antigen recognition. *Nat Mach Intell* 2024;6:1344–58. 10.1038/s42256-024-00913-8

[75] Singhaviranon S, Dempsey JP, Hagymasi AT. et al. Low-avidity T cells drive endogenous tumor immunity in mice and humans. *Nat Immunol* 2025;26:240–51. 10.1038/s41590-024-02044-z 39789375 PMC11785530

[76] Qin R, Zhang Y, Shi J. et al. TCR catch bonds nonlinearly control CD8 cooperation to shape T cell specificity. *Cell Res* 2025;35:265–83. 10.1038/s41422-025-01077-9 40011760 PMC11958657

[77] Chen L-Y, Li Y-P. Uncertainty quantification with graph neural networks for efficient molecular design. *Nat Commun* 2025;16:3262. 10.1038/s41467-025-58503-0 40188130 PMC11972353

[78] Soleimany AP, Amini A, Goldman S. et al. Evidential deep learning for guided molecular property prediction and discovery. *ACS Cent Sci* 2021;7:1356–67. 10.1021/acscentsci.1c00546 34471680 PMC8393200

[79] Muttenthaler M, King GF, Adams DJ. et al. Trends in peptide drug discovery. *Nat Rev Drug Discov* 2021;20:309–25. 10.1038/s41573-020-00135-8 33536635

[80] Lahat D, Adali T, Jutten C. Multimodal data fusion: an overview of methods, challenges, and prospects. *Proc IEEE* 2015;103:1449–77. 10.1109/JPROC.2015.2460697

[81] Steyaert S, Pizurica M, Nagaraj D. et al. Multimodal data fusion for cancer biomarker discovery with deep learning. *Nat Mach Intell* 2023;5:351–62. 10.1038/s42256-023-00633-5 37693852 PMC10484010

[82] The fourth paradigm: data-intensive scientific discovery. 2009.

