# Supplementary material for: A free energy perturbation-assisted machine learning strategy for mimotope screening in neoantigen-based vaccine design
Source: Brief Bioinform. 2025 Jul 10;26(4):bbaf254. doi: 10.1093/bib/bbaf254 (PMC12240735; doi:10.1093/bib/bbaf254)
Supplement: SuppData_bbaf254 [file suppdata_bbaf254.pdf]

A Free Energy Perturbation-Assisted Machine Learning Strategy for Mimotope Screening in Neoantigen-based Vaccine Design

Qinglu Zhong [a,b], Kevin C. Chan [a,b #], Lei Fu [a,b], Ruhong Zhou* [a,b,c,d]

[a] College of Life Sciences, College of Physics, Institute of Quantitative Biology, Zhejiang University, Hangzhou 310058, China

[b] Shanghai Institute for Advanced Study, Zhejiang University, 799 Dangui Road, Shanghai 201203, China

[c] Zhejiang Key Laboratory of Cell and Molecular Intelligent Design and Development, Zhejiang University, Hangzhou 310058, China

[d] Department of Chemistry, Columbia University, New York, NY 10027, United States

* To whom correspondence should be addressed: rhzhou@zju.edu.cn (R.Z.)

[#] Present address: Department of Biosciences and Bioinformatics, School of Science, Xi'an Jiaotong-Liverpool University, Suzhou 215123, China

Supplementary Figures

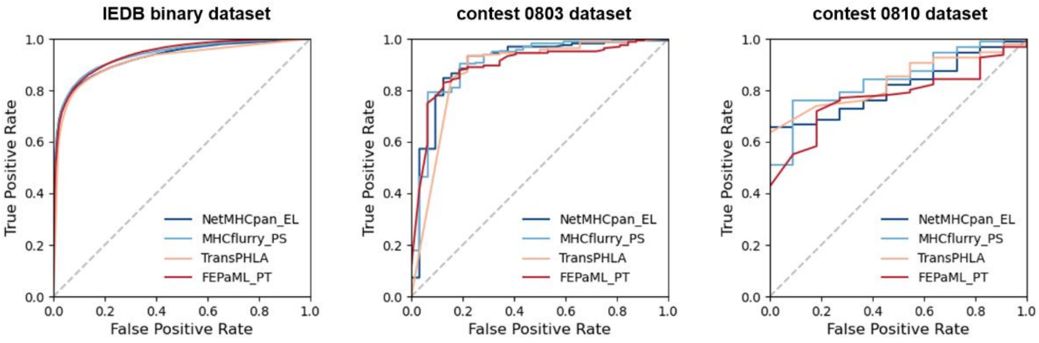

Figure S1. Receiver operating characteristic (ROC) curves of binding binary classification correspond to the results in **Figure 3A** of the main text.

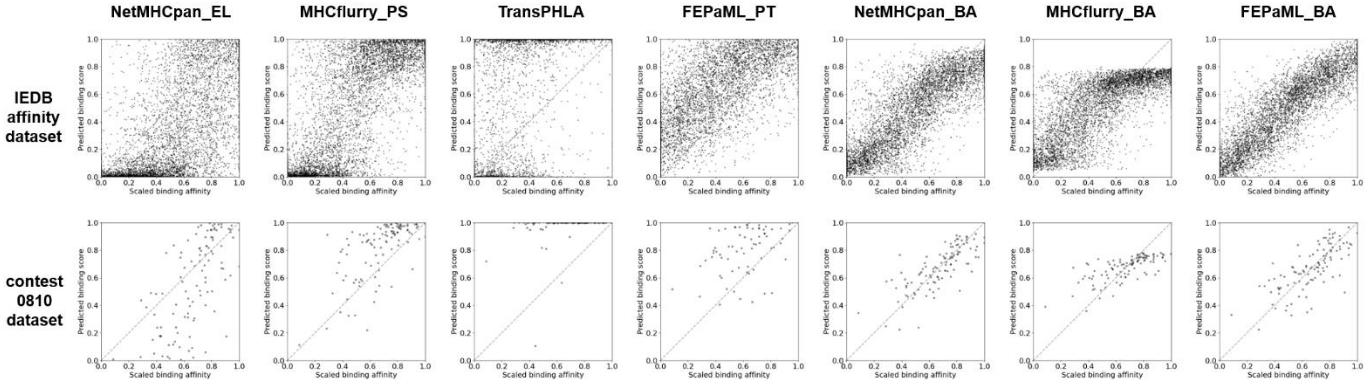

Figure S2. Scatter plots of binding affinity regression correspond to the results in **Figure 3B** of the main text. Binding affinity is scaled to a 0-1 binding score using the formula $1 - \log_{50000}(nM)$, where *nM* is the concentration unit associated with binding affinity. This transformation is acknowledged in ML processing. There are no scatter plots for the p53-mut FEP dataset because the dataset records relative binding free energy data rather than absolute binding affinity data. See scatter plots for the p53-mut FEP dataset in **Figure 4** of the main text.

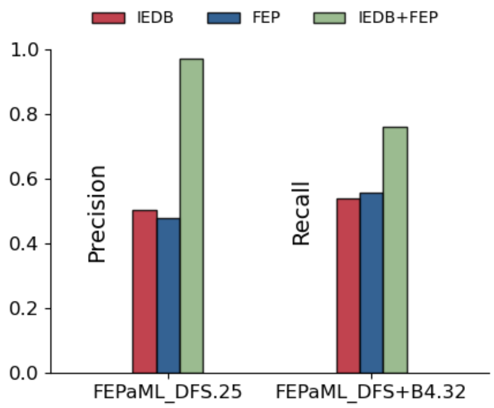

Figure S3. Ablation analysis of FEPaML_DFS.25 and FEPaML_DFS+B4.32 on p53-mut FEP dataset, corresponding to their optimal performance in **Figure 3C**.

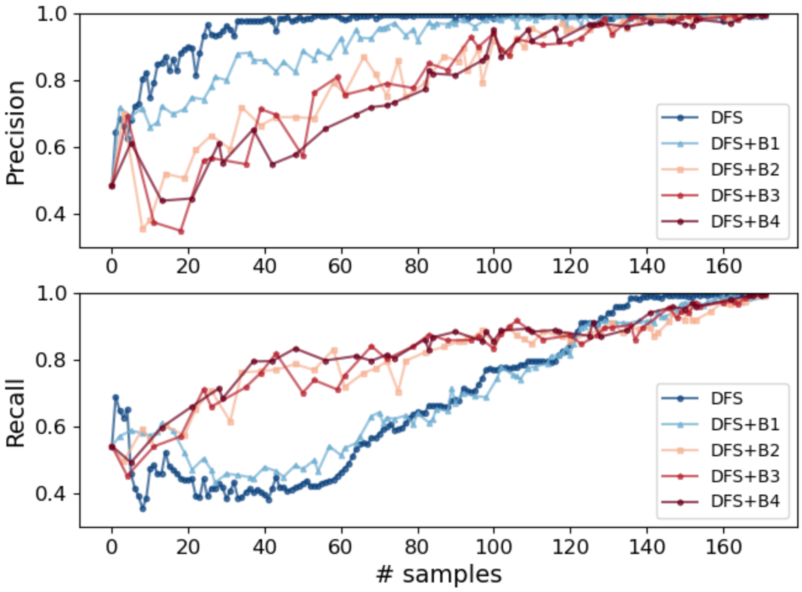

Figure S4. Precision (upper) and recall (lower) on NY-ESO-1-mut FEP dataset. X-axis is the number of FEP samples used for fine-tuning the ML model along with Bayesian optimization iteration. DFS+B*n* refers to Depth-First-Search combined with search Breadth of *n*.

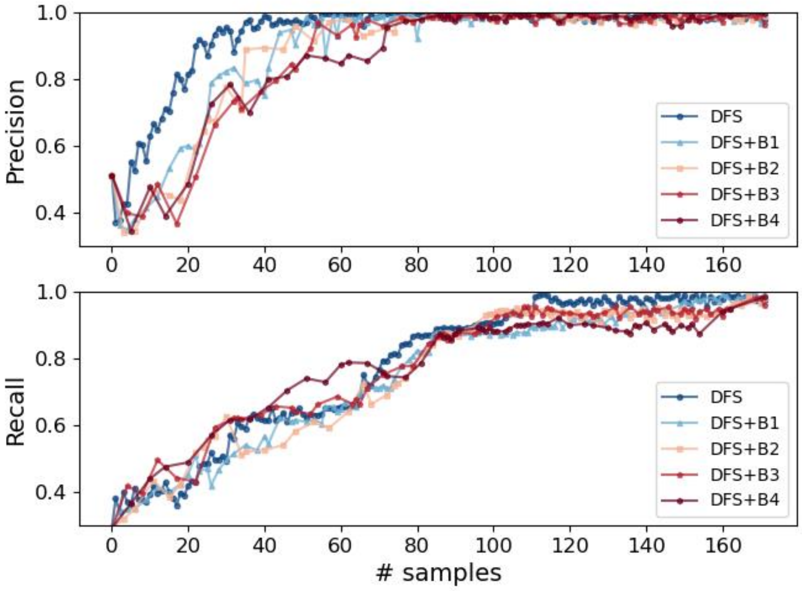

Figure S5. Precision (upper) and recall (lower) on WT1-mut FEP dataset. X-axis is the number of FEP samples used for fine-tuning the ML model along with Bayesian optimization iteration. DFS+B*n* refers to Depth-First-Search combined with search Breadth of *n*.
